# When can stress facilitate divergence by altering time to flowering?

**DOI:** 10.1002/ece3.1821

**Published:** 2015-12-09

**Authors:** Crispin Y. Jordan, Dilara Ally, Kathryn A. Hodgins

**Affiliations:** ^1^ Department of ZoologyUniversity of British ColumbiaVancouverBritish ColumbiaV6T 1Z4Canada; ^2^ Institute of Evolutionary BiologyThe University of EdinburghKings Buildings, Ashworth Laboratories, West Mains RoadEdinburghEH9 3LFUK; ^3^ Bayer CropScience LP890 Embarcadero Drive.SacramentoCA95605; ^4^ School of Biological SciencesMonash UniversityBuilding 18Victoria3800Australia

**Keywords:** Assortative mating, drought, flowering time, herbivory, local adaptation, *Mimulus guttatus*, phenology, phenotypic plasticity, water stress

## Abstract

Stressors and heterogeneity are ubiquitous features of natural environments, and theory suggests that when environmental qualities alter flowering schedules through phenotypic plasticity, assortative mating can result that promotes evolutionary divergence. Therefore, it is important to determine whether common ecological stressors induce similar changes in flowering time. We review previous studies to determine whether two important stressors, water restriction and herbivory, induce consistent flowering time responses among species; for example, how often do water restriction and herbivory both delay flowering? We focus on the direction of change in flowering time, which affects the potential for divergence in heterogeneous environments. We also tested whether these stressors influenced time to flowering and nonphenology traits using *Mimulus guttatus*. The literature review suggests that water restriction has variable effects on flowering time, whereas herbivory delays flowering with exceptional consistency. In the *Mimulus* experiment, low water and herbivory advanced and delayed flowering, respectively. Overall, our results temper theoretical predictions for evolutionary divergence due to habitat‐induced changes in flowering time; in particular, we discuss how accounting for variation in the direction of change in flowering time can *either increase or decrease* the potential for divergence. In addition, we caution against adaptive interpretations of stress‐induced phenology shifts.

## Introduction

Gene flow tends to homogenize populations, which reduces the potential for local adaptation (Lenormand 2002), speciation (Coyne and Orr 2004), and range expansion (Kirkpatrick and Barton 1997). In general, high migration relative to the strength of selection and genetic drift prevents population differentiation (Yeaman and Otto 2011, reviewed by Lenormand 2002). Migration of an allele from an environment in which it is favored to one in which it is disfavored causes migration load in the latter, which can generate selection for traits that reduce gene flow (Lenormand 2002). Similarly, numerous forms of assortative mating can evolve that reduce gene flow and promote diversification (e.g., Doebeli and Dieckmann 2003), including mating within groups due to differential timing (Antonovics 2006) or location of reproduction (Otto et al. 2008), and self‐fertilization (e.g., Dickinson and Antonovics 1973; Epinat and Lenormand 2009). In this light, studying mechanisms that reduce gene flow is fundamental to understanding the maintenance and generation of biodiversity.

The time to first flowering often changes when plants grow in different environments (reviewed by Levin 2009; also see below), and the possibility that an environmentally mediated phenological shift could facilitate the evolution of assortative mating by habitat type (e.g., habitats with different dominant stressors) has attracted increasing attention. Stam (1983) modeled this possibility, where a habitat‐induced shift in date of first flowering (HISF) causes habitat‐specific assortative mating. Specifically, he considered a population consisting of two patches that were identical, except for an environmental difference that induced an initially small, neutral, nongenetic (i.e., phenotypically plastic) change in flowering time between patches, for example, causing patch *A* to flower slightly before patch *B*, but the environmental difference has no effect on the duration of flowering by individuals; as well, no seed dispersal occurred between patches. The patches initially overlapped in flowering time, and genetic variation for flowering time existed in both patches, so that genes for “early" and “late" flowering were initially present in each patch. He showed that HISF caused the early flowering patch (*A*) to tend to receive pollen with alleles for early flowering from the later‐flowering patch (*B*); likewise, patch *B* tended to receive pollen with alleles that cause late flowering from *A*. Thus, HISF caused biased gene flow between patches for flowering time that alone caused genetic divergence for flowering time between the patches and reduced gene flow. Counterintuitively, simulations showed that increasing pollen dispersal between patches aided divergence in flowering time (a form of character displacement), which becomes obvious when one considers that no genetic divergence in flowering time could occur if there were no pollen migration between patches in this model. In contrast, seed dispersal between patches eroded genetic differences in flowering, independently of flowering time. Soularue and Kremer (2012) drew similar conclusions, using a quantitative genetics approach along an environmental cline.

Recent work has extended Stam (1983)'s study. Gavrilets and Vose (2007) showed that a small, environmentally induced phenological shift between habitats (on the order of the effect of a single gene‐substitution) greatly improved the opportunity for genetic divergence of flowering time between habitats, and Winterer and Weis (2004) examined how stress‐induced phenology shifts can affect the evolution of resistance to the stressor. Finally, Levin (2009) reviewed empirical cases of HISF, and proposed that habitat‐specific flowering times could result from plasticity alone, or a combination of plasticity and subsequent genetic differentiation. The theoretical expectation (Fox 2003; Weis et al. 2005; Devaux and Lande 2008) and empirical demonstration that within‐population variation in flowering time can cause assortative mating (Ennos and Dodson 1987; Weis and Kossler 2004) supports the argument that HISF should promote assortative mating by habitat type. Empirical studies have shown that genetic differences in flowering time have evolved among plant habitats, apparently reducing gene flow (e.g., Savolainen et al. 2006, reviewed by Antonovics 2006), and it is possible that HISF could have aided this process.

Real plant populations experience more complex environments than considered in the models, above. In particular, populations may be heterogeneous for multiple stressors, where each stressor may affect plant traits differently and influence opportunities for evolutionary divergence. For example, opportunities for divergence via HISF depend strongly on whether two stressors affect the *direction of change* in flowering time similarly. If two adjacent subpopulations experience different stressors that both shift flowering in the same direction (e.g., both shift toward earlier flowering relative to “benign" conditions), this similarity in the direction of flowering response can “cancel out" phenology differences between subpopulations. Thus, opportunities for evolutionary divergence between the subpopulations decrease relative to the case where only one subpopulation experiences a stressor (i.e., as considered by Stam 1983). In contrast, opportunities for evolutionary divergence can increase when the stressors shift flowering in opposite directions, because minor within‐patch phenology changes can produce large between‐population differences in flowering time. Hence, the consistency among stressors for the direction of flowering time shifts may prove a critical factor determining opportunities for evolutionary divergence via HISF in heterogeneous environments.

To better understand opportunities for evolutionary divergence by HISF, we used two approaches to study how two exemplary stressors, water restriction and herbivory, affect the direction of change in flowering time via phenotypic plasticity; we focussed on these two stressors due to their common occurrence, large effects on plant populations (Hawkes and Sullivan 2001), and availability of data from previous studies. We used a full‐sib design to examine how low water and herbivory affect time to flowering and other ecologically important fitness correlates (height and number of flowers produced) for similar genotypes of *Mimulus guttatus*. We also searched the literature to address the larger question, “how frequently do low water and herbivory change flowering time in the same direction (e.g., toward earlier flowering)?". The answer makes predictions for how often we expect HISF to impede or facilitate evolutionary divergence via a change in date of first flowering. This question of how often two stressors change flowering time in the same direction is implicitly descriptive, and can be addressed by a literature review, so long as the survey is not strongly biased (for example, by excessively representing a particular taxonomic group).

To address this question we ideally need to know how individual species respond to different stressors, yet few studies examine a species' flowering response to both low water and herbivory (or any particular combination of stressors, generally). Therefore, to interpret our descriptive approach we must assume that flowering responses observed among species reflect responses within species. For example, if herbivory and low water tend to delay and advance flowering times among species, respectively, then we assume that individual species will also tend to delay and advance flowering in response to these stressors, in which case HISF may facilitate divergence among subpopulations that differ in these stressors. Similarly, throughout our discussion we assume that phenology differences between subpopulations will affect gene flow (and the potential for evolutionary divergence, sensu Stam 1983; Soularue and Kremer 2012; see above) because we do not measure gene flow, directly. Our data clearly show that low water and herbivory commonly shift flowering in the same direction as well as in opposite directions, suggesting that HISF may commonly increase or decrease the potential for evolutionary divergence between subpopulations. Throughout, we follow previous convention that a “stressful" environment is one that decreases fitness (e.g., Fowler and Whitlock 2002; Armbruster and Reed 2005).

## Methods

### Production and maintenance of genetic lines

Our experiment used plants collected from the Wreck Beach population of *M. guttatus*, situated on the edge of the University of British Columbia campus. *Mimulus guttatus* displays showy yellow flowers and occurs as either an herbaceous annual (Hall and Willis 2006) or perennial. Our population has a perennial habit with observable vegetative reproduction through runners and seed production occurring through a mixture of selfing and outcrossing (selfing rate ≈59%, Ritland and Ganders 1987). This population occupies a sandy slope, with many plants growing in ground moistened by water fed from above.

In early summer of 2008 we collected a total of 38 plants on two sampling dates (32 and six plants), spaced at least 1 m apart to limit the sampling of genetically identical individuals produced by vegetative growth. Sampling on the two dates occurred in different areas of the population. We potted these plants in standard potting soil and watered them in the glasshouse as needed. Within each sampling date we randomly assigned individuals to mating pairs, with one member of the pair serving as the sire and the other as the maternal plant to produce 19 full‐sib lines.

We began crosses in late May 2008. All maternal flowers were emasculated in the bud phase, and freshly opened flowers were chosen on sire plants whenever possible; flower pedicels were marked with either a tag or liquid paper. We rubbed open anthers onto a stigma using tweezers until the stigma closed (Ritland and Ritland 1989), and wiped the tweezers between pollinations. We monitored each pollination and re‐applied pollen from the same donor on later dates if fruiting had not initiated. As our population readily sets fruit by autonomous selfing (C. Y. Jordan, D. Ally, K. A. Hodgins, pers. obs.), we occasionally removed excess fruits to aid maturation of our pollinated flowers; we collected fruits when they began to dehisce. All flowers remained uncovered throughout the experiment; however, unwanted pollination by pollinators was unlikely because we only noted three pollinators in the glasshouse over the course of a year.

### Growth of experimental plants

The experiment began in mid‐October, 2008. We chose approximately 40 filled seeds randomly from each maternal plant (unfilled seeds are unlikely to germinate; Searcy and Macnair 1990). We sowed full‐sibs together in single small pots, using a separate pot for each mother's seeds. The seeds germinated and grew for about 1 month, with the pots arranged randomly on a mist‐bench.

After about 4 weeks (November 12) we randomly assigned 10, 5 and 5 seedlings from each seed family to control (C), water‐stress (WS) and herbivory (H) treatments (described below), respectively; we used fewer individuals when germination rates limited seedling availability. Seedlings were transplanted individually into a 10 × 10 × 10 cm pot filled with standard potting mix. We randomly assigned pots to trays (≤10 pots per tray) with the restrictions that all plants in a tray belonged to the same treatment and that each tray contained only one member from any seed family. The plants were then allowed to recover from their transplant in the mist‐bench for 1 week; on November 20 all trays were moved to the main glasshouse area, where they received unfertilized water, delivered by hose. After 2 weeks in the glasshouse (December 5), we watered all plants except those in the WS treatment by flooding the bench for 7 min with fertilized water; beginning the following week, all plants were automatically watered every morning by this method (except WS; see treatment details below). All plants in this experiment received daylight, supplemented with glasshouse lights set for 16‐h days. We moved tray positions randomly within the glasshouse and plant positions haphazardly within trays approximately every 3 days until the beginning of January, 2009; beyond this time we gradually increased the time between randomization, to a maximum of once per week.

### Treatment descriptions

The WS treatment began 2 weeks after plants were moved to the main glasshouse area (see above), and the H treatment after 3 weeks (December 11). WS plants were raised several inches above the flooding bench so they generally experienced the same glasshouse conditions as the other plants. We lowered the WS plants onto the bench for watering by flooding when approximately 50% of WS plants began to wilt. The frequency of WS watering changed as the plants developed; WS plants received water in the same manner as there other treatments approximately once every 6 days early in the experiment, and once every 3 days toward the end.

Plants assigned to the H treatment experienced artificial herbivory once per week: we cut every new leaf greater than 26 mm in diameter in half (perpendicular to the main vein) with a pair of scissors, so every leaf was cut once. We cleaned the scissors with ethanol between cutting each plant. In addition, every week we sprayed the top and bottom of leaves of every H plant with a 1 mmol/L solution of methyl jasmonate, a ubiquitous plant compound that triggers biosynthetic pathways in response to wounding and herbivory (Doughty et al. 1995). This general method is used widely as a surrogate for natural herbivory (e.g., Agrawal et al. 1999; Cipollini and Sipe 2001).

The levels of stress imposed in each treatment were informed by pilot studies, and chosen to be strong enough to affect fitness, as measured by growth and flower production, but weak enough to minimize mortality. The intensity of the herbivory treatment likely matched previous studies: Carr and Eubanks (2002) found that spittlebug herbivory decreased flower production in *M. guttatus* by about 20%, similar to our results (see Results). In contrast, the intensity of our water‐stress treatment is likely lower than *M. guttatus* experiences in nature. For example, transplant experiments in the wild show that water stress often kills perennial *M. guttatus* before they flower (e.g., Hall and Willis 2006) (water stress in natural *M. guttatus* populations can occur as late‐season drought, whereas our treatment applied low water over a longer period). By minimizing mortality but reducing fitness in both stress treatments, we tried to apply low water and herbivory treatments that cause comparable intensities of stress. Had we applied water stress and herbivory with greatly different intensities (e.g., allowing one stressor to cause more mortality than the other), then it would have been unclear whether different flowering responses to low water versus herbivory were due to the nature versus the intensity of the stressors. While our approach helps to standardize our stressor strength, it also yields results consistent with previous studies (see Results).

Our use of fertilized water confounds water and nutrient stress, to some degree. However, we expect that water and nutrient limitation to occur simultaneously in natural populations because physiological studies suggest that water stress reduces nutrient uptake (Hsiao 1973; Chapin 1991). In addition, in the field, the transport of nutrients to roots can decrease in dry soil, and slowed root growth from water stress can reduce exploration of new soil for nutrients (Hsiao 1973). Hence, all water‐stress experiments likely confound water and nutrient limitation to some degree. Moreover, had we attempted to maintain nutrient levels between treatments, differences in water abundance would have changed the pH as well as the concentration of solutes and likely created osmotic stress, which then would be confounded with drought. Hence, it is difficult to isolate the effect of water availability, alone, on plant performance. Finally, a plant's physiological response to water, osmotic stress and nutrient deprivation are not independent, as all alter ABA, which in turn can affect key genes, such as transcription factors like bZIP and MYP that mediate a plant's response (Seki et al. 2002; Kang et al. 2009; Cramer et al. 2011; Para et al. 2014 (see figure 2)).

### Data collection

We checked our plants every 5 or 6 days to determine when each plant initiated flowering. At the end of the experiment (the week of March 7, 2009), we counted the number of fruits and flowers initiated by each plant and measured height (cm) after straightening. At this time, all plants showed signs of senescence and flowering had largely ceased: 52%, 22% and 48% of plants in the C, WS and H treatments, respectively, still displayed at least one open flower. For analyses, we calculated days to first flowering from the date that all plants were transplanted (November 12).

### Literature search

We searched the literature to determine how frequently water stress and herbivory cause earlier (or later) flowering among species. To identify relevant articles we conducted a Web of Knowledge search using the terms “flowering time" and “water stress" or “drought" or “herbivory." We excluded crop species due to the concern that their breeding histories may have included artificial selection with respect to water stress or herbivory, and complicated interpretation of the results. We also excluded studies that examined forms of herbivory that only involved damage to flowers, such as bud‐clipping by weevils (e.g., Ashman et al. 2004). We expanded our search using Google Scholar and by introducing the terms “phenology" and “plasticity".

All included studies met several criteria, described next. All studies demonstrated that water stress and herbivory significantly decreased some component of fitness (i.e., the treatments caused “stress"), with two exceptions. First, Franks (2011) did not study conventional fitness components but focused on water‐use efficiency (WUE), and found higher WUE in a low water treatment. Second, Agren and Schemske (1993) found only marginally significant effects of herbivory on fitness (flower production, *P* = 0.07), but noted specifically that their design had low power to detect such effects. All studies needed to indicate the direction of change in flowering time; we also noted when studies did not control for genetic background (Table S1). Some studies did not test whether a stressor affected flowering time or fitness in an appropriate way for our analysis (e.g., perform relevant contrasts among treatments); therefore, we included those studies where it was possible to use *t*‐tests to test for these effects, using appropriate means, SE's and degrees of freedom, based on data provided in an article's printed text or Tables (see Table S1). We estimated the magnitude of flowering time effects from figures when they were not reported directly in tables or text. Magnitudes of flowering shifts, on their own, however, do not clearly illustrate the potential for divergence, which also depends on other metrics (Elzinga et al. 2007). For example, a ten‐day shift in flowering will have a greater impact on divergence in a species that flowers for 30 days than when flowering lasts 100 days. Hence, because most studies do not report on aspects of flowering (e.g., the duration of flowering) other than its initiation, we present magnitudes of flowering shifts for illustrative purposes, only. We noted the life history of species, which was usually indicated in the published papers; when it was not, we confirmed the life history using the USDA plant database or we contacted the authors.

### Data analysis

#### *Mimulus* glasshouse experiment

We analyzed our data with the MCMCglmm package [version 2.10; (Hadfield 2010)] in R (version 2.12.1). MCMCglmm uses Markov chain Monte Carlo routines to fit generalized linear mixed models in a Bayesian framework. All analyses used expanding priors, which are typically uninformative and facilitate sampling of parameter space by helping to avoid chains becoming stuck at certain values (J. Hadfield, personal communication). Significance is assessed by the posterior distribution of the model's parameters. MCMCglmm allows treatments to have different residual variances when analyzing interactions in mixed‐effects models. We emphasize that standard likelihood‐based mixed‐effects models (e.g., lme package in R) produced similar results to those reported here, using MCMCglmm. We tested whether each stressor affected traits relative to the control treatment, this comparison being of most biological interest.

We fitted mixed‐effects models, with treatment fitted as a fixed effect and line as a random effect. We modeled Line x Treatment (LxT) interactions with a constant correlation/covariance structure, which considers equal genetic variance among treatments and allows correlation for a genotype's response to all treatments, but assumes this correlation is consistent among treatments. All data were ln‐transformed to help meet the standard assumptions for Gaussian distributed mixed‐effects models.

Some plants experienced damage due to handling during the experiment (e.g., when randomizing positions). When damage affected measurements of height or the total number of flowers produced we omitted damaged plants from the analyses. All combinations of 19 lines and three treatments had at least three individuals even after removing damaged plants from the dataset. For the smallest dataset, the mean number of plants per line × treatment combination equaled 7.9, 4.6 and 4.4 for the C, WS and H treatments, respectively.

## Results

### *Mimulus* glasshouse experiment

Almost all plants flowered during our experiment. Among the original 371 plants, we only excluded 12 plants from flowering time analyses (five, three and four individuals from the control, herbivory and water‐stress treatments, respectively) because of mortality before flowering, failing to flower, or damage. Given such weak selection on the probability of flowering, any shifts in time to first flower among the treatments must have been due to phenotypic plasticity, and not due to changes in allele frequencies among the treatments.

Time to flowering varied greatly within each treatment. We calculated the mean time to flowering per Line‐Treatment combination; the variance in these means equaled 10.5, 14.7 and 15.4, for the C, WS, and H treatments, respectively, and did not differ significantly among treatments (Levene's test, df = 2, *F* = 0.434, *P* = 0.65). These variance measures characterize the broad variation in flowering time within all treatments; for example, the difference between the latest and earliest recorded date of first flowering within the C, WS, and H treatments equaled 32, 27 and 27 days, respectively. Given this variation, our coarse sampling interval (5 or 6 days) is adequate to detect flowering time shifts caused by experimental conditions (see below).

**Figure 1:**
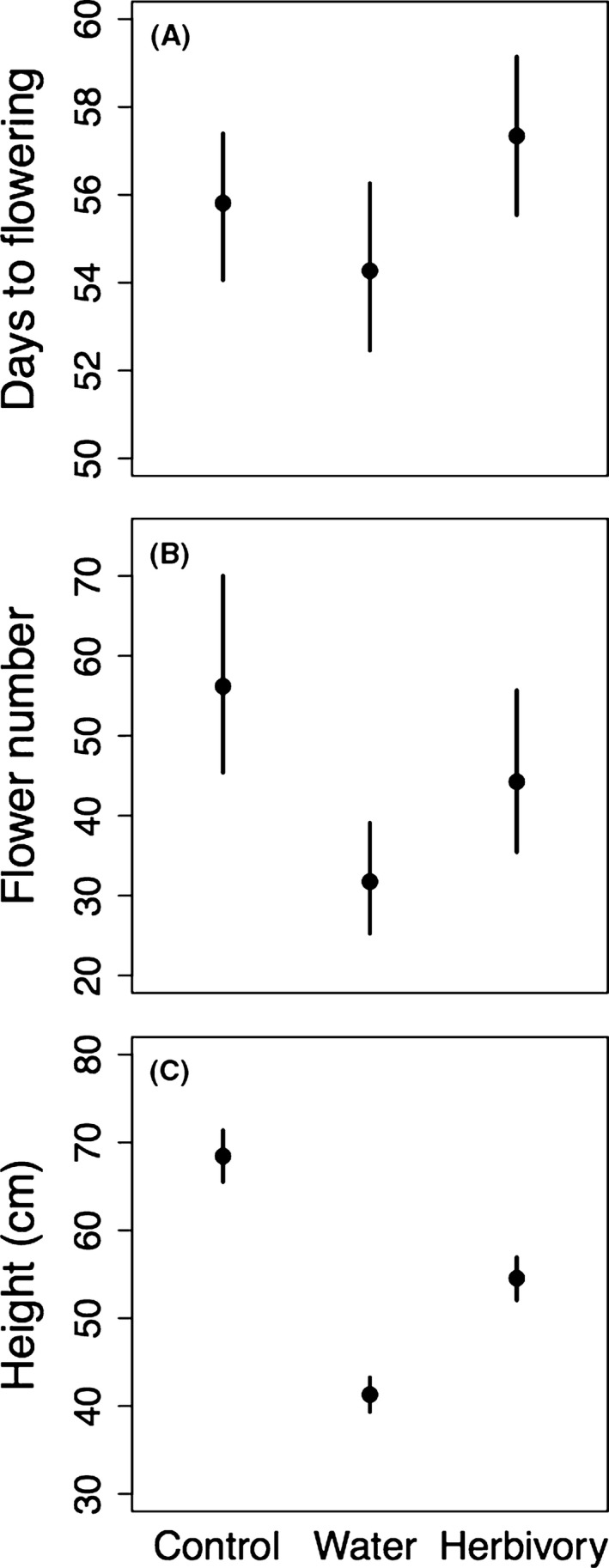
Responses of plants from 19 genetic lines of *Mimulus guttatus* to water stress and herbivory, as measured by (A) number of days to flowering, (B) total number of flowers produced, and (C) height. Error bars represent 95% confidence intervals; asymmetric CI's result from back‐transformation of the data. Estimates are produced from mixed‐effects models that include a Line x Treatment interaction. All comparisons between treatment effects and the control are significant, except for the contrast between the water stress treatment and the control for days to flowering, which is marginally significant (see text).

Water stress caused flowering to occur 1.5 days earlier compared to the control (Fig. 1A), which was marginally significant (*P* = 0.061) in a model that included a LxT term. However, there was no evidence in this model for a Line‐by‐Treatment interaction, as the 95% highest probability density interval of the posterior distribution included zero. Removing the LxT term from the model, the effect of water stress on flowering time is significant (*P* < 0.05). In contrast, simulated herbivory delayed flowering by approximately 1.5 days relative to the control, and this effect was significant whether or not the nonsignificant LxT term was included (*P* < 0.05 and *P* < 0.01, respectively). The flowering response to water stress is extremely similar to previous studies using *M. guttatus* (see Table S1), suggesting that differences in experimental conditions among studies had little impact on flowering time responses among the studies. On the other hand, we detected a (small, 1.5 day) significant change in flowering time under herbivory when a previous study of *M. guttatus* failed to do so (Ivey et al. 2009; Table S1), suggesting that even our coarse sampling was sufficient to detect flowering time shifts.

Both stressors reduced flower production. Compared to the control, water stress reduced flower production by approximately 45% (Fig. 1B), which was significant both in models that included and omitted the nonsignificant LxT term (*P* < 0.001 in both models). Likewise, the herbivory treatment reduced flower production by 21% relative to the control (Fig. 1B), in models that included or excluded the nonsignificant LxT term (*P* < 0.05).

Stress also reduced the height of plants in both stress treatments. Plants that experienced the water‐stress and herbivory‐treated plants were approximately 27 and 14 cm shorter (40% and 21%) than plants in the control treatment, respectively (both stressors significantly different from the control; *P* < 0.001 for models that include or exclude the nonsignificant LxT term; Fig. 1C). Reduced flower production and height suggest that our low water and herbivory treatments caused *M. guttatus* stress.

### Literature review

We qualitatively analyzed our literature review; we considered trends in directional shifts in flowering time when responses were statistically significant or not, noting that interpretation is clearer in the former case.

Our literature review yielded a phylogenetically diverse dataset both within and among stressors. We identified 44 studies (including this one) that document flowering time responses of 22 and 12 species to water stress and herbivory, respectively; in total, these include 31 species from 12 families. Within each stressor, almost all species belong to different genera; *Mimulus* is the only genus represented by more than one species within a stressor (water stress: *M. guttatus*,* M. nasutus*,* M. nudatus*). Hence, the sampled species tend to be relatively distantly related within stressors. Species composition is slightly less phylogenetically independent between the stressors: three species (*Brassica rapa*,* M. guttatus*,* Sinapis arvensis*) were used in both water stress and herbivory experiments; in addition, the genus *Lotus* was represented in both treatments (*Lotus corniculatus* and *Lotus wrangelianus* in water stress and herbivory, respectively). Hence, among 31 species sampled, three are present in both treatments; among the 12 families represented, five are represented in both stressors.

The available data suggest that the direction of shift in time to first flowering tends to change consistently within species in response to a given stressor, despite variation in experimental conditions among independent tests. Consider the eight species (four in each of the herbivory and low water treatments) that experienced a given stressor multiple times in independent tests *and* yielded more than one statistically significant response to the stressor (Table S1) (for example, *Hordeum spontaneum* exhibited statistically significant delayed flowering in two independent tests of low water, and *Ipomopsis aggregata* displayed statistically significant flowering delays in eight independent tests of herbivory). Among these that experienced herbivory, all four species (*Campanulastrum americanum*,* Chamaecrista fasciculata*,* I. aggregata*,* Raphanus raphanistrum*) always exhibited (statistically significant) delays in flowering in independent tests within (and among) species; this consistency in response within species occurred when different forms (e.g., deer vs. clipping) or intensities (e.g., 25 vs. 50% leaf removal) of herbivory were applied to the focal species (Table S1). Similarly, the (statistically significant) direction of flowering change was similar *within* three species (*Eriogonum abertianum*,* H. spontaneum*,* M. guttatus*) that experienced low water in multiple independent tests (Table S1). The fourth species, *B. rapa*, exhibited statistically significant earlier and delayed flowering in response to low water (Table S1) among different studies. However, the experimental conditions differed greatly between the experiments that yielded these contrasting results for *B. rapa*: the study that found significantly earlier flowering (Franks et al. 2007; Franks and Weis 2008) used seeds from natural populations and measured flowering time as days between an individual's germination and first flower production, whereas the study that found delayed flowering (Steinbrenner et al. 2012) used seeds from the University of Wisconsin's Fast Plants program (http://www.fastplants.org) and examined the percent of individuals that had begun flowering at 18 days postsowing.

Among species, water stress tended to elicit earlier flowering almost as frequently as it did delayed flowering. Among the 13 species with statistically significant responses to water stress, five flowered significantly earlier and seven later (and *B. rapa* responded in both directions; Table S1). Similarly, among the 16 species that had statistically nonsignificant flowering time responses, water stress elicited (nonsignificant) earlier flowering for 10, delayed flowering in three, and both (nonsignificant) responses in three. Given that almost all species belong to separate genera, phylogenetic constraints would need to extend deep within a group's phylogeny to drive the observed equal representation of responses.

Flowering responses to herbivory were highly consistent among species. Among the 11 species that exhibited statistically significant flowering responses to herbivory, all significant responses involved delayed flowering (Table S1). Nonsignificant flowering responses occurred for four species; among these, trends toward delayed flowering occurred exclusively in two species (Table S1; *Anthemis cotula*,* B. rapa*), and both (nonsignificant) earlier and delayed flowering was recorded in separate tests for each of two other species (Table S1; *L. wrangelianus*,* R. raphanistrum*). As above, given that all species here belong to unique genera, it is unlikely that phylogenetic constraints underlie the consistent response to herbivory. That said, the lack of variation in response to herbivory makes it impossible to test for phylogenetic constraint in this flowering response.

## Discussion

### Phenological shifts and differentiation: directionality

In environments with a single stressor, the magnitude of a habitat‐induced shift in flowering time (HISF) and other characteristics of flowering (e.g., duration of flowering) affect opportunities for evolutionary divergence (Elzinga et al. 2007). In heterogeneous environments, however, the direction of flowering shifts becomes crucial for divergence, because small shifts in mean flowering times within subpopulations can be magnified among subpopulations when stressors in different subpopulations shift flowering times in opposite directions. Likewise, parallel changes in the direction of flowering shifts can erode phenology differences among subpopulations, even when large flowering shifts occur within subpopulations. Our literature review and experimental data with *M. guttatus* suggest that flowering responses to water stress and herbivory can both positively and negatively influence opportunities for divergence, sensu Stam (1983) and Soularue and Kremer (2012) (see Introduction). We focus on the initiation of flowering by necessity (other data are scarce), and encourage studies to examine other aspects of phenology (Elzinga et al. 2007); also, our approach ignores additional effects of stress on a plant's biology, such as how a change in height or flower production affects interactions with pollinators, and thereby potentially alters gene flow.

By reviewing literature for flowering responses to two important stressors, we implicitly assume that trends found among species will apply within species, on average (see Introduction). Among species, herbivory showed a strong tendency to delay flowering, whereas low water tended to elicit earlier and delayed flowering with relatively equal frequency. If these same trends manifest within species, we expect that individual species will very frequently (say, roughly half of the time) delay flowering in response to both low water and herbivory, but many others will shift their dates of first flowering in opposite directions in response to these stressors – these predictions apply most to species with similar qualities to those in our dataset, i.e., largely temperate species with (mostly) annual life histories and a phylogenetic distribution similar to our dataset. For species that shift the date of first flowering in the same direction for both stressors, we therefore predict that opportunities for evolutionary divergence via HISF diminish in heterogeneous environments compared to environments with a single stressor, because the phenology shifts effectively cancel each other out. This prediction remains unchanged when additional aspects of flowering phenology change under stress (e.g., flowering duration), although characters such as flowering duration may still promote divergence (Fox 2003; Elzinga et al. 2007). On the other hand, when water stress and herbivory affect flowering time in opposite directions, as occurred in our *Mimulus* experiment, we expect that HISF will facilitate evolutionary divergence. Specifically, phenotypically plastic changes in date of first flowering (HISF) in response to a stressor may bias gene flow with respect to flowering time, and lead to at least partial reproductive isolation (via phenology) between subpopulations with different stressors (Stam 1983; Soularue and Kremer 2012). Overall, these findings suggest that HISF may often (but not always) facilitate evolutionary divergence, highlight the species‐specific nature of this potential for divergence, and temper predictions for the role of HISF in population divergence (Stam 1983; Gavrilets and Vose 2007; Levin 2009).

Life history appears unrelated to the observation that herbivory exclusively delays flowering whereas low water has variable effects on date of first flowering. First, annual species dominate the dataset so that a specific life history is unlikely to cause apparent differences in flowering responses between the stressors (likewise, our predictions, generally, apply most to annuals). Second, annuals and perennials tended to elicit similar responses within each stressor. For instance, among the four perennial species that were tested with water stress, two displayed significantly delayed flowering (*L. corniculatus* and *Lobelia siphilitica*, a short‐lived perennial), one flowered significantly earlier (*M. guttatus*), and the fourth, *Lychnis flos‐cuculi*, had a nonsignificant response toward earlier flowering (both flowering responses appear similarly displayed by annual species; Table S1). Furthermore, both annual and perennial varieties of *M. guttatus* flowered significantly earlier under water stress (Table S1). Third, the two stressor datasets comprised relatively similar proportions of perennial species: four of 22 species (including *M. guttatus*) and four of 12 species were perennials that experienced low water and herbivory, respectively (Fisher exact test, *P* = 0.45). Therefore, stressor type is not greatly confounded with life history.

### Is HISF adaptive?

Herbivory delays phenology with exceptional consistency (see also Tiffin 2000). This occurred despite the use of many forms of “herbivory" among the surveyed studies, which can affect plants differently (Strauss and Agrawal 1999). This flowering behavior might involve nonadaptive causes; for example, reduced resources or consumed meristems may require resource or meristem replenishment before flowering can begin (e.g., *I. aggregata*, Juenger and Bergelson 1998, 2000; but see Brody and Irwin (2012), where resource addition did not affect phenology when combined with herbivory). If a HISF‐induced flowering delay is adaptive, it may be an “escape" strategy: several studies show that seed predation is highest at peak flowering (reviewed by Elzinga et al. 2007). Therefore, if herbivory at one time reliably indicates the probability of seed predation (or consumption of flowering shoots) in the near future, selection might favor delayed flowering to escape seed or flower loss. If true, we predict that the magnitude of delayed flowering will be largely independent of levels of experimentally induced herbivory. Among the studies in our review, only one (Hanley and May 2006) can appropriately test this hypothesis and, counter to expectations, greater “herbivory" (cotyledon removal) intensity increased the delay in flowering; further study will clarify when delayed flowering due to herbivory can function as an escape strategy.

Water stress caused either advanced or delayed flowering, among species. Earlier flowering might serve to partially escape water stress (e.g., Franks et al. 2007; Lovell et al. 2013), by achieving some reproductive fitness before water becomes too limiting. Similarly, delayed flowering may aid resistance or tolerance of water stress (Strauss and Agrawal 1999). However, we caution against such adaptive interpretations, particularly for water stress: if HISF is adaptive, we predict that the direction of HISF will match the evolution of flowering time in response to a stressor. In some cases this holds true. For example, *B. rapa* evolved earlier flowering in response to drought (Franks et al. 2007), and also advanced its phenology in low water experimental conditions (Franks et al. 2007; Franks and Weis 2008; but see Steinbrenner et al. 2012). Similarly, for herbivory, *Oenothera biennis* evolved earlier flowering in experimental plots with suppressed insects (Agrawal et al. 2012), consistent with expectations from Table S1. In contrast, *H. spontaneum* and *Triticum dicoccoides* evolved earlier flowering in recent decades, presumably due to aridization (Nevo et al. 2012), yet both species delayed flowering under experimental low water treatments (*H. spontaneum*: Volis et al. 2002, 2004; Nevo et al. 2012; *T. dicoccoides*: Nevo et al. 2012). The mismatch between the evolutionary change versus behavior in low water experiments might reflect inadequacy of experimental conditions to reflect the environment where evolution occurred. For instance, Steyn et al. (1996) suggest that similar stressors can elicit either advanced or delayed phenology, depending on the time of year the experiment is carried out; that said, our limited data (see Results) suggest that focal species respond similarly to a given stressor in independent tests, so that flowering responses to a stressor might be robust to some environmental differences (see further discussion, below). Alternatively, HISF may not be adaptive, but reflect constrained responses to the environment. Further studies of the potential adaptive significance of plastic flowering shifts are needed.

### Phenological shifts and differentiation: effect size

In general, plant populations connected by gene flow are more likely to diverge genetically when the environment induces large differences in flowering time among habitats, increasing variance in time to flowering, as a whole (Stam 1983; Gavrilets and Vose 2007; Levin 2009). The magnitude of phenology shifts in our experiment were, however, small (and almost identical to previous studies; Table S1; Murren et al. 2006; Ivey et al. 2009; Wu et al. 2010; Ivey and Carr 2012). Both stressors shifted flowering time by approximately 1.5 days relative to the control; given that flowering spans more than 2 months among our experimental plants, this shift in flowering time represents a small reduction of overlap in flowering between treatments. Furthermore, despite exhibiting signs of senescence, at least 22% of individuals displayed open flowers within each treatment at the time we harvested the plants (see Methods); hence, differences among treatments for the termination of flowering are unlikely to greatly increase phenology differences among our *Mimulus* treatments.

Effect sizes seen in our experiment are also common in our literature review (Table S1). For example, if we consider statistically significant responses to stressors (where larger effect sizes are expected), water stress and herbivory change the date of first flowering (on average, among species) by 5.7 and 7.7 days, respectively (although flowering durations are typically not reported to provide context to these values). Of course, some large effect sizes do sometimes occur (e.g., 26 days; Table S1), where opportunities for divergence seem more likely. For illustration, phenological variation among alpine plant populations due to differential timing of snow melt can produce a positive correlation between flowering time differences and genetic divergence (with a maximum $F_{ST}\approx 0.2$ for 30 days of separation between *Veronica stelleri* populations), whereas no correlation occurs for between‐population distance and genetic divergence over the spatial scale analyzed (up to 3 km between patches) (Kudo 2006). Overall, given that theory suggests that even a small environment‐induced phenological change can facilitate differentiation (e.g., of the magnitude of the effect of a single gene‐substitution; Gavrilets and Vose 2007), empirical tests are needed to clarify the biological significance of the observed phenological shifts (and other aspects of phenology; e.g., flowering duration, Elzinga et al. 2007) for subsequent divergence in flowering time (see Weis and Kossler (2004) for potential methods).

### Consistency of response to stressors

So far, we have assumed in our Discussion that phenology shifts in response to a stressor occur consistently within a species (see Results for data consistent with this assumption). It is difficult to know whether this assumption holds for either stressor. With respect to herbivory, on one hand, the high consistency of flowering responses within and among species supports our assumption. For example, with eight separate tests of *I. aggregata*' s response to herbivory, one can use a binomial test to show that it is unlikely that all eight responses would involve delayed flowering by chance. On the other hand, some tests of *I. aggregata* are not independent because they study the same population, so the results may not be representative of the entire species. With respect to water stress, statistically significant responses to low water within a species might reflect that species' “typical” response to low water, but too few data are available to test whether any species responds consistently to low water. Also, it is possible that variation in flowering responses to low water within and among species, to some extent, results from variation in experimental conditions. If true, then our primary conclusion (that HISF may aid or deter genetic divergence in heterogeneous environments) would remain unchanged, but it would apply more appropriately on a population level than a species level. For example, if the conditions of water stress (e.g., intensity or timing) differ among populations, and if these differences cause variation in the direction of change in flowering time among populations, then the potential for HISF to drive evolutionary divergence will simply vary among populations.

## Conclusions

Studies that consider the role of stress in evolution have traditionally addressed its influence on phenotypic and genotypic variance (e.g., Stanton et al. 2000; Fowler and Whitlock 2002). Stress‐induced changes in flowering time present another mechanism for stress to promote evolution in heterogeneous environments via assortative mating. Assortative mating, in general, is likely important for plant evolution (Ennos and Dodson 1987; Fox 2003; Weis and Kossler 2004; Winterer and Weis 2004; Weis et al. 2005). The current results suggest that the potential for divergence between subpopulations via HISF depends on how stressors affect the direction of phenology shifts, and temper conclusions by previous studies that promote phenotypic plasticity in flowering time as a means to facilitate evolutionary divergence (e.g., Stam 1983; Gavrilets and Vose 2007; Levin 2009). Our conceptual approach considered the simple scenario where subpopulations each experience unique primary stressors; future field studies that investigate (i) how multiple stressors within subpopulations interact to affect phenology, (ii) the biological significance of small differences in flowering time between stressful and benign environments, and (iii) the effect of stress on nonphenological phenotypes for gene flow (e.g., how stress affects a plant's attractiveness to pollinators), will clarify how stressors influence divergence.

It is intriguing to note that a number of species best known to have evolved reproductive isolation over short distances and to display different flowering times between habitats (e.g., *Anthoxanthum odoratum*, Antonovics 2006; *Howea* spp., Savolainen et al. 2006) are wind‐pollinated; as wind‐pollinated species may have narrower flowering periods than animal‐pollinated species (Rabinowitz et al. 1981), they may be more prone to HISF‐facilitated divergence. Whether characteristics of wind‐pollinated species (e.g., extent of pollen dispersal, relatively short duration of flowering) make wind‐pollinated species more susceptible to divergent evolution for flowering time between environments would be a fascinating subject for future studies.

## Conflict of Interest

None declared.

## Supporting information


**Table S1.** Effect of stressors on time to first flower for various species.
