# Supplementary material for: When can stress facilitate divergence by altering time to flowering?
Source: Ecol Evol. 2015 Dec 9;5(24):5962–73. doi: 10.1002/ece3.1821 (PMC4717339; doi:10.1002/ece3.1821)
Supplement: Supplementary file 1 — Table S1. Effect of stressors on time to first flower for various species. [file ECE3-5-5962-s001.docx]

Table S1: Effect of stressors on time to first flower for various species. For Low Water and Herbivory stressors, life history (A, Annual; B, Biennial; P, Perennial; SLP, Short-Lived Perennial) follows species name and is listed once per species, unless we found multiple life histories occurred for a species. All shifts in flowering time refer to a difference between a control (benign) treatment and the given stressor; -, +, and ns indicate earlier, later, and non-significant changes in flowering date, respectively. "0" indicates identical mean dates of first flowering between benign and stress treatments. '.' indicates identical flowering time under stressful and benign conditions ("Phenology Shift") or data not available in source article ("Change (Days)").

| Stressor | Species | Phenology Shift | Change (Days) |
| --- | --- | --- | --- |
| WATER STRESS |  |  |  |
| Low water | *Arabidopsis thaliana*^1^ (A) | - (ns) | unknown |
| Low water | *Arabidopsis thaliana*^2^  Epigenetic RILS | + (ns) | 0.2 |
| Low water | *Arabidopsis thaliana*^2^  Control lines | . (ns) | 0.0 |
| Low water | *Avena barbata*^3,4,*^ (A) | - | 8 |
| Low water | *Brachypodium distachyon*^5,*^(A)  (desert pop.) | - (ns) | 1.2 |
| Low water | *Brachypodium distachyon*^5, *^  (mediterranean pop.) | - | 5.1 |
| Short vs. long wet season | *Brassica rapa*^6,7,*^ (A)  Ancestral, wet population | -^a^ | 5.4 |
| Short vs. long wet season | *Brassica rapa*^6,7,*^  Descendent, wet population | -^a^ | 3.9 |
| Short vs. long wet season | *Brassica rapa*^6,7,*^  Ancestral, dry population | -(ns) | 0.2 |
| Short vs. long wet season | *Brassica rapa*^6,7,*^  Descendent, dry population | .(ns) | 0.0 |
| Low water | *Brassica rapa*^8^ | + (ns) | ~1 |
| Early, temporary drought, nutrients | *Brassica rapa*^9,*^ | + | .^b^ |
| Low water | *Bromus fasciculatus*^5,*^ (A)  (desert pop.) | - (ns) | 1.5^c^ |
| Low water | *Bromus fasciculatus*^5,*^  (mediterranean pop.) | - (ns) | 1.4^c^ |
| Low water | *Chaetanthera moenchioides*^10,*^ (A) | - | ~7 |
| Low water, nutrients | *Dimorphotheca sinuata*^11,*^ (A)  Sown in April | + (ns) | 5 |
| Low water, nutrients | *Dimorphotheca sinuata*^11,*^  Sown in May | - (ns) | 6 |
| Simulated drought | *Eriogonum abertianum*^12,*^ (A) | +^d^ | . |
| Low water | *Eriogonum abertianum*^12^ | +^d^ | . |
| Low water | *Erucaria hispanica*^5,*^ (A)  (desert pop.) | - (ns) | 0.9 |
| Low water | *Erucaria hispanica*^5,*^  (mediterranean pop.) | - (ns) | 1.9 |
| Low water, nutrients | *Foveolina albida*^11,*,e^ (A)  Sown in April | + (ns) | 13 |
| Low water, nutrients | *Foveolina albida*^11,*,e^  Sown in May | - | 9 |
| Low water | *Hordeum spontaneum*^13^ (A) | + | 7.3^f^ |
| Low water | *Hordeum spontaneum*^14^ | + | 6.2 |
| Low water | *Impatiens capensis*^15^ (A) | - (ns) | ~1 |
| Low water | *Lobelia siphilitica*^16^ (SLP) | + | 4 |
| Low water | *Lobelia siphilitica*^16^ | + (ns) | ~3 |
| Low water | *Lobelia siphilitica*^16^ | + (ns) | ~2 |
| Low water at high CO_2_, 18°C | *Lotus corniculatus*^17,g^ (P) | + | ~10 |
| Low water, 18°C | *Lotus corniculatus*^17,g^ | - (ns) | ~1 |
| Low water (periodic drought) | *Lychnis flos-cuculi*^18^ (P) | - (ns) | 3 |
| Low water, nutrients | *Mimulus guttatus*^19^ (P) | - | 1.5 |
| Low water, nutrients | *Mimulus guttatus*^20^ (A) | -^e^ | 1.1 |
| Low water | *Mimulus guttatus*^21^ (A) | -(ns) | ~2 |
| Low water | *Mimulus guttatus*^22^ (P) | -(ns)^a^ | 2.25 |
| Low water | *Mimulus guttatus*^22^ (A) | -(ns)^a^ | 0.96 |
| Low water | *Mimulus nasutus*^22^ (A) | -(ns)^a^ | 0.24 |
| Low water | *Mimulus nudatus*^22^ (A) | +(ns)^a^ | 0.2 |
| Low water | *Nigella degenii*^23^ (A) | + | 9-16 |
| Low water, nutrients | *Senecio arenarius*^11,*^ (A)  Sown in April | + | 5 |
| Low water, nutrients | *Senecio arenarius*^11,*^  Sown in May | - (ns) | 7 |
| Low water | *Sinapis arvensis*^24^ (A) | - (ns)^a^ | 0.1 |
| Low water, nutrients | *Ursinia cakilefolia*^11,*^ (A)  Sown in April | + | 2 |
| HERBIVORY |  |  |  |
| Herbivory (insecticide and molluscicide) and low competition | *Anthemis cotula*^25, *^ (A) | + (ns) | ~3 |
| Herbivory (insecticide  and molluscicide)  and med. competition | *Anthemis cotula*^25,*^ | + (ns) | ~2 |
| Herbivory (insecticide  and molluscicide) and high competition | *Anthemis cotula*^25,*^ | + | ~3 |
| Herbivory (caterpillar) | *Brassica rapa*^26^ (A) | + (ns) | ~0.1 |
| Herbivory (caterpillar) | *Brassica rapa*^27,h,i^ | + | ~4^f^ |
| Herbivory (deer) in light gap | *Campanulastrum americanum*^28,*^ (A, B) | + | ~16 |
| Herbivory (clipping) in light gap | *Campanulastrum americanum*^28,*^ | + | ~12 |
| Herbivory (deer) in understory | *Campanulastrum americanum*^28,*^ | + | ~11 |
| Herbivory (clipping) in understory | *Campanulastrum americanum*^28,*^ | + | ~9 |
| Herbivory (25% leaf removal) | *Chamaecrista fasciculata*^29,*^ (A) | + | 5^b^ |
| Herbivory (50% leaf removal) | *Chamaecrista fasciculata*^29,*^ | + | 6^b^ |
| Herbivory (insect) | *Citrullus lanatus*^30,*^ (A) | + | ~7 |
| Herbivory (clipping) | *Ipomopsis aggregata*^31,*^ (P) | + | 8.8 |
| Herbivory (clipping) | *Ipomopsis aggregata*^31,*^ | + | 9.3 |
| Herbivory (clipping) | *Ipomopsis aggregata*^32,*^ | + | 7 |
| Herbivory (clipping) | *Ipomopsis aggregata*^32,*^ | + | 14 |
| Herbivory (clipping) | *Ipomopsis aggregata*^33^ | + | ~6 |
| Herbivory (natural damage) | *Ipomopsis aggregata*^33^ | + | ~2 |
| Herbivory (clipping) | *Ipomopsis aggregata*^34,*^ | + | 26 |
| Herbivory (clipping) | *Ipomopsis aggregata*^35,*^ | + | ~18 |
| Herbivory (cotyledon removal) | *Leontodon hispidus*^36,*^ (P) | + | ~20 |
| Herbivory (insect removal) | *Lotus wrangelianus*^37,j,*^ (A) | + (ns) | ~14 |
| Herbivory (insect removal) +Competitor | *Lotus wrangelianus*^37,j,*^ | - (ns) | ~10 |
| Herbivory (insect removal) | *Lotus wrangelianus*^37,k,*^ | + (ns) | ~6 |
| Herbivory (insect removal) +Competitor | *Lotus wrangelianus*^37,k,*^ | + (ns) | ~12 |
| Herbivory (clipping leaves, adding methyl-jasmonate) | *Mimulus guttatus*^19^ (P) | + | 1.5 |
| Herbivory (insect) | *Mimulus guttatus*^38^ (A) | . (ns) | 0 |
| Herbivory (caterpillar) | *Raphanus raphanistrum*^39^ (A) | + | 3 |
| Herbivory (caterpillar) | *Raphanus raphanistrum*^40^ | + | 2.8 |
| Herbivory (caterpillar) | *Raphanus raphanistrum*^41^ | - (ns) | ~0.4 |
| Herbivory (caterpillar) | *Raphanus raphanistrum*^41^ | + (ns) | ~1 |
| Herbivory (caterpillar); ½ each leaf removed; glasshouse | *Raphanus raphanistrum*^42^ | + (ns) | 1.2 |
| Herbivory (caterpillar); ¼ each leaf removed; glasshouse | *Raphanus raphanistrum*^42,i^ | - (ns) | 0.1 |
| Herbivory (caterpillar); ½ of each of first 4 leaves removed; glasshouse | *Raphanus raphanistrum*^42^ | + (ns) | 0.7 |
| Herbivory (caterpillar); ½ each leaf removed; growth chamber | *Raphanus raphanistrum*^42^ | - (ns) | 0.5 |
| Herbivory (caterpillar); ½ of each of first 4 leaves removed; growth chamber | *Raphanus raphanistrum*^42^ | + (ns) | 0.1 |
| Herbivory (Groundhog) | *Sinapis arvensis*^43,*^ (A) | + | 3-7 |
| Herbivory (insect) | *Solidago altissima*^44,l^ (P) | + | unknown |

1 Pigliucci et al. (1995); 2 Zhang et al. (2013); 3 Sherrard and Maherali (2006); 4; Sherrard et al. (2009); 5 Aronson et al. (1992); 6 Franks et al. (2007); 7 Franks and Weis (2008); 8Franks(2011);9Steinbrenneretal.(2012);10Bull-Heren ̃uandArroyo(2009);11Steyn et al.(1996); 12 Fox (1990); 13 Volis et al. (2002); 14 Volis et al. (2004); 15 Heschel and Riginos (2005); 16 Caruso (2006); 17 Carter et al. (1997); 18 Hauser and Loeschcke (1996); 19 This study; 20 Murren et al. (2006); 21 Ivey and Carr (2012); 22 Wu et al. (2010); 23 Jorgensen and Andersson (2005); 24 Stanton et al. (2000); 25 Erneberg (1999); 26 Strauss et al. (1999); 27 Agren and Schemske (1993); 28 Lin and Galloway (2010); 29 Frazee and Marquis (1994); 30 Biernacki and Lovett-Doust (2002); 31 Juenger and Bergelson (1997); 32 Juenger and Bergelson (1998); 33 Juenger and Bergelson (2000); 34 Freeman et al. (2003); 35 Brody and Irwin (2012); 36 Hanley and May (2006); 37 Lau and Strauss (2005); 38 Ivey et al. (2009); 39 Agrawal et al. (1999); 40 Strauss et al. (1996); 41 Lehtila ̈ and Strauss (1997); 42 Lehtila ̈ and Strauss (1999); 43 Cipollini and Sipe (2001); 44 Meyer and Root (1993)

^a^ Significance of flowering time inferred from t-tests that used ls-means and SE

^b^ Use cumulative % flowers open by given date; shift in flowering date based on median flowering times

^c^ Phenology assessed as time of bolting stage

^d^ Failure time analysis

^e^ Article contains a typo: correction confirmed with author.

^f^ Mean response over multiple treatments/populations, estimated from figure.

^g^ Only analyzed one genotype.

^h^ Effects averaged over 2 trichome number treatments.

^i^ Unclear from results whether stressor significantly reduced fitness.

^j^ Year = 2002

^k^ Year = 2003

^l^ herbivory effect found under high but not low soil fertility; data analysis did not include 'genotype'; do not report flowering date, but only % plants that had produced inflorescence buds by a given date.

^*^ Study does not replicate genetic families among treatments.
